# Peptic ulcer characteristics in oral opium and non-opium user patients with upper gastrointestinal bleeding

**DOI:** 10.1186/s12876-024-03137-7

**Published:** 2024-01-22

**Authors:** Mohsen Masoodi, Mohammad Sabzikarian, Nikta Masoodi, Saeed Farhadi, Gholam Reza Rezamand, Seidamir Pasha Tabaeian, Atefeh Talebi, Farimah Fayyaz

**Affiliations:** 1 Colorectal research center, Iran University of medical sciences, Tehran, Iran; 2 Colorectal research center, Hazrat Rasoul Medical Complex, Niayesh Street, Sattarkhan Avenue, Tehran, 1445613131 Iran

**Keywords:** Gastrointestinal bleeding, Opium, Addiction, Ulcer, Endoscopy

## Abstract

**Background/Aims:**

Upper gastrointestinal bleeding (UGIB) is a frequent medical issue. The primary risk factors for bleeding peptic ulcers are Helicobacter pylori infection and non-steroidal anti-inflammatory drugs. The association between acute gastric/duodenal ulcer and opium use has been previously proposed; however, there is no available data on endoscopic findings of patients with acute UGIB who use opium.

**Materials and methods:**

In the present descriptive cross-sectional study, endoscopic data of 50 consecutive patients with oral opium use and 50 consecutive patients without any opium use who were admitted for UGIB were recorded. The size (5–10 mm, 11–20 mm, or more than 20 mm), number (single, double, or multiple), and location of the ulcers (esophagus, gastric corpus including the fundus and body, antrum, angulus, or duodenum) were examined by endoscopy in both groups.

**Results:**

Three or more ulcers were observed in 46% and 16% of patients with oral opium use and without opium use, respectively (*P*-value = 0.001). The rate of giant ulcers (> 20 mm) was significantly higher in patients who used oral opium (40% vs. 12%; *P*-value = 0.007). Esophageal ulcers were also more common in oral opium users (30%) than non-users (8%) with UGIB (*P*-value = 0.01). Nevertheless, the location of the ulcers between the two groups generally was not statistically different.

**Conclusions:**

This study has demonstrated that multiple, large peptic ulcers in GIB are potential complications of oral opium use. This could aid the needed modifications in the treatment protocol for these patients.

## Introduction

Upper gastrointestinal bleeding (UGIB) is a frequent medical issue resulting in high medical care costs and morbidity [1–3]. The annual hospitalization rate for acute UGIB is reported to be approximately 67 per 100,000 adults in the United States [4]. It presents with hematemesis or/and melena [5]. The most common causes of UGIB are ulcers of the stomach and duodenum, gastritis or duodenitis, esophagogastric varices, and erosive esophagitis [6]. While previous studies suggested that peptic ulcer disease was responsible for approximately half of UGIBs, more recent studies indicate that it is currently a less common cause [7–10]. Despite the availability of treatment to eradicate helicobacter pylori (H. pylori), the UGIB remains a prevalent complication. The reason behind this is the increased use of aspirin and non-steroidal anti-inflammatory drugs (NSAIDs) [11].

Opiates are found naturally in poppies such as morphine and codeine. Opiates are mainly used to treat pain; however, they are illegally inhaled or taken orally for nonmedical purposes. Although opium consumption has GI side effects, including nausea, vomiting, dyspepsia, dysmotility, and constipation, there is a disagreement over the effect of opium on gastric mucosa and GI ulcer development [12–17]. A significant association between acute gastric/duodenal ulcer and opium use has been demonstrated previously [15]; nevertheless, data on endoscopic findings of patients with acute UGIB who use opium is not available. The present study aimed to compare the endoscopic findings of patients with UGIB who used opium orally to those who did not report opium use.

## Materials and methods

In the present descriptive cross-sectional study, among patients with UGIB treated in Rasoul-e-Akram Hospital from January 2017 to December 2019 who had at least one ulcer in their endoscopy, 50 consecutive patients with oral opium use and 50 consecutive patients without any opium use were included. The sample size was calculated using the formula as follows [18, 19]:

$$ n \geqslant 2\frac{{{{\left( {{z_{1 - \alpha /2}} + {z_{1 - \beta }}} \right)}^2}{\sigma ^2}}}{{{{\left( {{\mu _1} - {\mu _2}} \right)}^2}}} $$

The α is error type I, β is error type II, and $$ \begin{array}{*{20}{c}}{\begin{array}{*{20}{c}}{\frac{{{\mu _1} - {\mu _2}}}{\sigma }} \end{array}} \end{array}$$ is effect size, and they are considered 0.95, 0.20, and 0.6, respectively. The sample size was calculated to be 50 in each case and control group.

Patients with a previous UGI operation for peptic ulcer or its complications, including ulcer perforation and bleeding, recent acute UGIB, advance liver disease, GI malignancy, renal impairment, Zollinger-Ellison syndrome, previous use of proton-pump inhibitors and H2-blockers in the last three months, ingestion of steroids, anticoagulant, salicylates or NSAIDs, history of alcohol use, smoking, pregnancy and lactating women were excluded from the study. We excluded patients using NSAIDs based on history alone in both groups at admission and at discharge, despite some limitations of this method.

The demographic data (age, gender), clinical information (opium, NSAIDs, and other drug histories, the type, and duration of symptoms), and endoscopic findings of the included patients were recorded under the supervision of a researcher gastroenterologist. The patients have a history of continuous opium use and a urine test for opiates was performed to confirm the opiate use status in patients.

All patients had received intravenous pantoprazole and were referred to experienced gastroenterologists for UGI endoscopy within 24 h of admission. Gastric biopsy samples were taken for rapid urease testing (RUT) to diagnose H. pylori.

Gastric and duodenal ulcers were defined as mucosal defects with 5 mm or more diameter, and ulcers larger than 20 mm in diameter are considered giant ulcers [20]. In endoscopy, the size of the ulcers (5–10 mm, 11–20 mm, or more than 20 mm), the number of ulcers (single, double, or multiple), and the location of the ulcers (esophagus, gastric corpus including the fundus and body, antrum, angulus, and duodenum) were examined in both groups. If endoscopy showed multiple ulcers, the size of the largest ulcer was recorded. Ulcer size was measured by a standard 5 mm fully open spoon endoscopic sampling forceps.

### Informed consent

was obtained from patients, and the local ethical committee approved the study.

### Statistical analyses

The results were statistically described as Means ± SD in continuous variables and frequency and percentage in categorical variables. The Chi-square and Fisher exact tests were used to evaluate the association between categorical variables, including number, size, and site of ulcers and the use of oral opium. The level of statistical significance was considered to be 0.05. The SPSS version 24.0 was used for statistical analysis.

## Results

A total of 100 patients met the study’s entry criteria and provided informed consent to register. Fifty patients with UGIB had oral opium use (Group 1), and 50 patients with UGIB had no opium use (Group 2). In group 1, the mean duration of oral opium use was 11.55 ± 4.37 years, and the median daily amount of oral opium ingestion was 1.12 ± 0.36 g. All patient in study was non-medical user of opium. Median frequency of use was 1–2 time per day. The opiate types used by patients in the study are raw opium (teriak) that is the air-dried extract of the opium poppy plant. The possible concentration was 100% of extract, but it is not possible for us to carry out a detailed examination on the opium samples of the patients. The mean age of group 1 patients was 53.04 ± 14.14 years, and for group 2, it was 48.46 ± 17.60 years. Forty-two patients in group 1 and 30 patients in group 2 were males. Patient with exclusion criteria not included in study for example 6 opium user and 5 non opium user excluded from study that use PPI previously. The patients’ characteristics are presented in Table 1.

**Table 1:** Patients’ characteristics in oral opium and non-opium user patients with upper gastrointestinal bleeding

		Non-opium user patients (*n* = 50)	Oral opium user patients (*n* = 50)	*P*-value
**Age; mean ± SD, year**		53.04 ± 14.14	48.46 ± 17.60	0.1546
**Gender; number (%)**	**Female**	8 (16%)	20 (40%)	0.008
	**Male**	42 (84%)	30 (60%)	
**H. pylori; number (%)**	**Positive**	41 (82%)	44 (88%)	0.556
	**Negative**	9 (18%)	6 (12%)	

In addition to the ulcer, erosive gastritis was observed in 42% of group 1 patients and 40% of group 2 patients. H. pylori-positive testing were observed in 82% of group 1 patients and 88% of group 2 patients.

The endoscopic findings (number, the largest diameter, and location of ulcers) are shown in Table 2.

In group 1 patients, one ulcer was seen in 16 (32%), two ulcers in 11 (22%), and three or more ulcers in 23 (46%) patients. Meanwhile, in group 2 patients, one ulcer was seen in 30 (60%), two ulcers in 12 (24%), and three or more ulcers in 8 (16%) patients. In oral opium addict patients, the maximum size of ulcers was 5–10 mm in 11 (22%), 11–20 mm in 19 (38%), and more than 20 mm in 20 (40%) patients. Whereas in non-opium user patients, it was 5–10 mm in 17 (34%), 11–20 mm in 27 (54%), and more than 20 mm in 6 (12%) patients. The number and size of ulcers were significantly higher in opium user patients with UGIB than in the other group. The rate of ulcers in the corpus, angulus, antrum and duodenum was relatively similar between the two groups. However, esophageal ulcers were more common in group 1 (30%) than in group 2 (8%). Nevertheless, the location of the ulcers between the two groups was not statistically different. Most esophageal ulcer in non-opium user seen on or near GEJ (3 near GEJ and one in lower third of esophagus) but ulcer in opium user seen also in other part of esophagus (7 on or near GEJ, 4 in lower and 4 in middle third of esophagus) but analysis need more sample size.

**Fig. 1:**
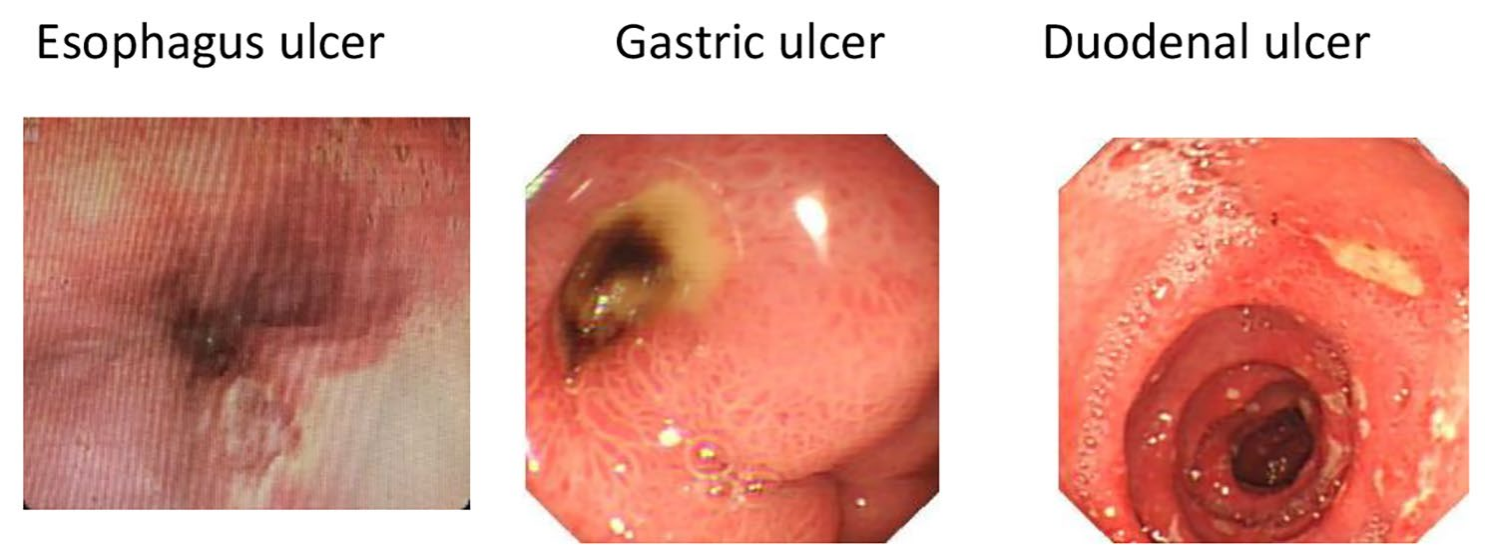
Esophageal, gastric and duodenal ulcers in oral opium users. Esophagus ulcer Gastric ulcer Duodenal ulcer

The endoscopic images of characteristic ulcers in patients with a history of opium use are presented in Fig. 1.

**Table 2:** Endoscopic characteristics in oral opium and non-opium user patients with upper gastrointestinal bleeding

		Oral opium user patients (*n* = 50)	Non-opium user patients (*n* = 50)	*P*-value	Total *P*-value
**Number of ulcers**	Single Ulcer	16 (32%)	30 (60%)	0.005	0.001
	Two ulcers	11 (22%)	12 (24%)	0.812	
	Three or more ulcer	23 (46%)	8 (16%)	0.001	
**Size of ulcers**	5–10 mm	11 (22%)	17 (34%)	0.181	0.007
	11–20 mm	19 (38%)	27 (54%)	0.108	
	> 20 mm	20 (40%)	6 (12%)	0.001	
**Site of ulcer**	Esophagus	15(30%)	4 (8%)	0.005	0.01
	Corpus	16 (32%)	14 (28%)	0.662	
	Angulus	7 (14%)	5 (10%)	0.538	
	Antrum	9 (18%)	7 (14%)	0.585	
	Duodenum	28(56%)	31(62%)	0.542	

## Discussion

Opium is one of the herbal remedies used to treat symptoms such as pain, diarrhea, or anxiety. The opioid is a generic term that refers to both opiates and their synthetic analogs derived from opium. Known adverse effects of the opioid on the GI tract include nausea, vomiting, inhibition of gastric emptying, dyspepsia, reduction of lower esophageal sphincter relaxation, abdominal distension, dysmotility, constipation, narcotic bowel syndrome, irritable bowel syndrome, spasm or dysfunction of the sphincter of Oddi, and common bile duct dilation [14, 16, 17]. Moreover, opium is a risk factor for gastric and other cancers [13, 21, 22]. Currently, opium is produced chiefly illegally and is used for nonmedical purposes. In 2017, it was estimated that 53 million (47–60 million) people worldwide, or 1.1% of the population between the ages of 15 and 64, were past-year opioid users [23]. In Asia, opioid use is prevalent, and 1% of the population has used opium at least once in the past year. In the Middle East and Southeast Asia, 2.3% of the adult population uses opioids. In the Islamic Republic of Iran, approximately 90% of opioid users reported the use of crude opium or condensed opium ash extracts [24]. Generally, the past-year use of opioids is much higher among men than women (4% vs. 0.2% of the population). However, the rate of opium use among women, especially young people, has increased in recent years [23]. In the present study, 84% of the opium users group were male. As there are no studies that show age- and sex-related ulcer size and number, this effect is difficult to discuss and requires further study, and it is more likely that this difference did not influence the conclusions.

It is generally believed that NSAIDs and H. pylori cause peptic ulcer disease. To control the confounding factor of the H. pylori-positive test in UGIB patients, the rate of H. pylori-positive patients was similar between the case and control groups. For rapid result we used the rapid urease test (RUT) to detect Helicobacter pylori in the case and control groups, and despite the possibility of false positive or negative results, it did not show a significant difference between the two groups as a risk factor for ulceration. Also, if the ulcer was suspected to be malignant, a biopsy was performed and the patients were excluded from the study with the result of cancer. Esophageal ulcer biopsy was also performed from ulcer, which excluded viral infection and allergic background. Also none of our patients had sign and symptoms of viral infection.

There are limited reports in the literature regarding the induction of GI ulcers by opium. Mahajan et al. proposed the term “opioid abuse gastroenteropathy” in young male patients who presented with either gastric outlet or small bowel obstruction due to opioid-induced ulcers and ulcerated strictures. Most of these cases responded poorly to medical management and endoscopic balloon dilatation, needing surgical intervention [12]. These findings may be due to decreased motility, elevated resting muscle tone, small intestine ischemia, and hyper inflammation effects of opium [25]. Moreover, Häuser et al. reported a case of colitis due to opium use [26].

Although morphine inhibits the inflammation in experimental rats, it does not induce ulceration of gastric mucosa due to its anti-inflammatory effects. However, morphine can induce vagal stimulation, increase histamine release, and potentiate the ulcerogenic activity of indomethacin by prostaglandin inhibition and reduction of mucosal defense. It has been demonstrated that opioid antagonists such as naloxone significantly reduced the intensifying effect of morphine on ulcerogenic side effects of indomethacin [27, 28]. In one study on risk factors of peptic ulcers, a significant association was observed between acute gastric ulcers (*P* = 0.022, OR = 2.823) and acute duodenal ulcers (*P* = 0.023, OR = 2.326) with opium use [13].

On the contrary, some studies showed the protective effect of opioids on gastric mucosa. An animal study reported that the use of morphine before stress induction protects the gastric mucosa against ulceration and accelerates stress ulcer healing. This effect may be due to the stimulation of gastric mucosa to produce prostaglandins, leading to increased gastric mucosal secretions [15]. In another study on rats, no manifestation of the promoting effect of morphine on ulceration was observed. In contrast, it was suggested that morphine had protective effects against GI mucosal damage induced by aspirin and taurocholic acid. Therefore, increased mucus barrier strength mediated by the cytoprotective activity of morphine was reported [29]. In addition, Gyires et al. demonstrated that morphine inhibited the gastric mucosal damage induced by ethanol in rats, and naloxone blocked the protective effects of morphine [30]. Kahron et al. reported that sudden withdrawal of opiates in addicted people might cause gastroduodenal perforation. Morphine could decrease the destructive effect of acid on the epithelium; therefore, the sudden discontinuation of opiates without maintenance therapy may lead to perforation in the gastroduodenal area following the damaging effects of pepsin and acid on sensitive gastric and duodenal mucosa [31]. There are conflicting findings on the effect of opioids on the secretion of gastric acid. However, the antiulcer effect of morphine is unlikely to be mediated by the suppression of gastric acid secretion [15, 32–35].

Lanas et al. showed that tramadol was not associated with an increased risk of UGIB in patients admitted for peptic lesion bleeding [36]. In another study on patients with perforated peptic ulcers, tramadol appeared to increase mortality due to the masking of perforation symptoms and increased surgical delay. However, tramadol is less likely to mask the symptoms of bleeding peptic ulcers; therefore, it did not increase the mortality of bleeding peptic ulcers [37]. Altogether, the findings of studies on the effect of opioid agonists on gastric mucosal damage were contradictory, and the need for further investigation is felt.

Multiple peptic ulcers and ulcers with atypical locations in patients with recurrent peptic ulcer disease indicate a hypersecretory syndrome such as gastrinoma or the use of NSAIDs [38, 39]. However, methamphetamine or cocaine use and chronic cigarette smoking could be additional risk factors [40, 41]. Multiple ulcers were reported in 2–31.8% of patients examined with a fiberoptic endoscope [1, 19, 40, 42–44].In the present study, 46% of oral opium users had three or more ulcers, indicating a significant ulcerogenic effect of oral opium.

A study on patients with H. pylori infection and peptic ulcer showed that the average size of duodenal ulcers was 9.3 ± 4.9 mm, and in gastric ulcers, it was 13.4 ± 7.5 mm. The average size of ulcers was 9.49 ± 5.3 mm in patients with the Forrest III type and 11.59 ± 6.8 in other types [19]. In the current study, 40% of oral opium users had ulcers larger than 20 mm. Our results indicated that the ulcers’ number and size were significantly higher in oral opium users. This may be due to opium or opium additives, including drugs, lead, clay, dyes, and rotten fruit [45–47].

In the present study, esophageal ulcers but not gastroduodenal ulcer were more common in oral opium user patients, which may be due to the adhesion of oral opium to the mucosa during consumption. Out of 15 opium-using patients with esophageal ulcer, 4 cases were in the middle third and 5 cases in the lower third, and 6 patients were at the gastroesophageal junction, and out of 4 non-opium-using patients with esophageal ulcer, one case was in the lower third and 3 patients were at the gastroesophageal junction. As a result, the proximal esophageal ulcer shows a higher number in opium users but analysis need more sample size.

Also, a few previous studies have demonstrated that opium consumption could be associated with higher rates of gastroesophageal reflux (GERD), which could be attributed to the effect of opium on decreasing GI motility [16]. In a large cross-sectional study in Iran, Islami et al. showed that opium use was associated with a 70% increase in the odds of severe GERD symptoms [48]. In another cross-sectional study in Iran, the rate of opium addiction was higher in patients with GERD (19.7% vs. 7.9%) [49]. Therefore, more ulcers at the gastroesophageal junction and esophagus in oral opium users may be due to more GERD in these patients.

The current study has potential limitations. The collection of the number and size of ulcers as categorical instead of continuous variables limited the statistical evaluation. Also, the composition of the opium mixture used by the patients is an essential factor that should be considered. The inaccessibility to the consumed opium was a limitation that needs to be addressed and explored in future studies. Another limitation of our study was the lack of patient follow-up and investigation of treatment response. Future studies are encouraged to investigate the impact of opium use on the treatment regimens of UGIB patients and to evaluate the treatment response by follow-ups.

To further evaluate the effects of opium on GI ulceration and bleeding, we need more pathophysiologic, population-based, and patient case studies.

## Conclusion

The present study has demonstrated that multiple large peptic ulcers and GI bleeding are complications of oral opium use. We recommend appropriate patient education regarding the potential adverse effects of oral opium use and withdrawal of opium in peptic ulcer cases.

### Main Points

- This is the first study on gastrointestinal bleeding and ulceration in opium users.
- We recommended appropriate patient education regarding the potential adverse effects of oral opium.

## Data Availability

The datasets generated during and analyzed during the current study are not publicly available due to the confidentiality of the participants and avoiding the re-identification of the cases, but are available from the corresponding author on reasonable request.
